# Surface electrical impedance myography detects disease in an adult-onset SOD1-G93A zebrafish model of amyotrophic lateral sclerosis

**DOI:** 10.1038/s41598-025-19830-w

**Published:** 2025-10-14

**Authors:** Seward B. Rutkove, Priyansh Shah, Laura Hevenor, Gaurav Tiwari, Dhrumil Patil, Tyler Mourey, Janice A. Nagy, Anjali K. Nath

**Affiliations:** 1 Department of Neurology, Beth Israel Deaconess Medical Center, Boston, MA 02115 USA; 2 Harvard Medical School, Boston, MA 02115 USA; 3 Department of Cardiology, Beth Israel Deaconess Medical Center, Boston, MA 02115 USA; 4 Zebrafish Core Facility, Beth Israel Deaconess Medical Center, Boston, MA 02215 USA; 5 Broad Institute, Cambridge, MA 02142 USA

**Keywords:** Amyotrophic lateral sclerosis, Neuromuscular disease, Skeletal muscle atrophy, Zebrafish, Preclinical animal models, Electrical impedance myography, Reliability measures, Biomarkers, Skeletal muscle, Slow twitch muscle fibers, Fast twitch muscle fibers

## Abstract

Amyotrophic lateral sclerosis (ALS) is a progressive neurodegenerative disease that is characterized by loss of motor neurons and atrophy of skeletal muscle. Current FDA-approved drugs to treat ALS are only modestly effective at slowing the progression of the disease. Rodents have been the standard preclinical animal model for testing candidate ALS drugs; however, alternative animal models, including zebrafish, are being studied to accelerate therapeutic discovery. Here, we sought to advance a model of ALS in zebrafish with associated tools to serve as biomarkers of neuromuscular deterioration. Thus, we applied noninvasive, surface electrical impedance myography (EIM) methodology to SOD1^G93A^ zebrafish and control animals to evaluate its ability to serve as an electrophysiological biomarker of disease in ALS zebrafish. Measurements were acquired from the caudal musculature of animals at 2 time points by applying an alternating current at 41 frequencies (1 kHz–1 MHz) and measuring the resulting voltages. At the first time point, SOD1^G93A^ animals still exhibited normal body morphometrics, spinal cord motor neuron numbers, and skeletal muscle mass, while at the second time point, these SOD1^G93A^ animals exhibited reduced weight, loss of motor neurons, type 1 and 2 myofiber atrophy, and decreased capacity for endurance swimming. We found that non-invasive surface EIM detected the alterations observed in diseased ALS zebrafish at the second time point. Specifically, EIM measurements (phase angle, reactance, and resistance) at 2 and 50 kHz were robust metrics that distinguished between healthy and diseased zebrafish. To assess the reliability of our EIM technique in healthy and ALS zebrafish, we calculated the intraclass correlation coefficient and conducted Bland–Altman analyses. The EIM methodology exhibited excellent reproducibility in both healthy and ALS zebrafish. In sum, these findings demonstrate that EIM is an effective tool to detect neuromuscular disease in symptomatic adult ALS zebrafish, and the approach described here offers a fast, noninvasive, and reliable platform that holds the potential to test candidate drug therapeutic efficacy.

**Supplementary Information:**

The online version contains supplementary material available at 10.1038/s41598-025-19830-w.

## Introduction

Amyotrophic lateral sclerosis (ALS) is a fatal neurodegenerative disease that can be classified into two major categories: heritable and sporadic. Among the genetic cases, the most common mutations are in *SOD1*, *TARDBP*, *C9ORF72*, and *FUS*^1–5^. Among the sporadic cases, the etiology is unknown. However, several environmental risk factors have been associated with ALS, including military service, and occupational exposure to pesticides and metals^6–10^. Irrespective of the cause, the final common pathway is characterized by motor neuron degeneration leading to muscle atrophy and paralysis, occasionally accompanied by cognitive impairment, with most patients dying of respiratory failure ~ 3–5 years after symptom onset^3,11,12^.

Studies using murine models of ALS have improved our understanding of the pathophysiology of ALS^13–15^. Yet some drugs tested in murine models, in which the results were interpreted as “promising,” ultimately failed in human ALS trials^14–17^. The unsuccessful clinical translation of potential therapeutic candidates has led to a call to the ALS research community for new models and tools to enable the screening and discovery of ALS therapies^14,18,19^. Consequently, alternative animal models of ALS are beginning to emerge to bridge the gap between drug discovery and clinical research^19,20^. One promising alternative model for ALS therapeutic discovery is the zebrafish^21^.

Zebrafish are an established organism to study neurodegenerative diseases, including Parkinson’s disease, Huntington’s disease, and others^22–26^. Zebrafish neurobiology has a particularly high degree of evolutionary conservation with humans at the genetic, molecular, cellular, and behavioral levels^27–30^. Concordantly, zebrafish neurodegenerative disease models exhibit the hallmark features of human brain pathology, including protein aggregation, synaptic dysfunction, motor neuron axonopathy, and loss of neuronal cells^21–26,31–35^. A robust repertoire of stereotypic behavioral phenotypes in zebrafish can be leveraged to assess motor function in neurogenerative zebrafish mutants^28^. Moreover, these small animals have an economy of scale that is not feasible in other vertebrate systems, in the sense that large numbers of animals can be maintained for long periods of time at relatively low cost. Finally, methodological advantages compared to rodent models include the feasibility of real-time, whole-organism imaging via microscopy, and their experimental tractability with an established toolbox for chemical and genetic perturbations in high throughput.

Genetic models of ALS in zebrafish have been generated by targeting *sod1*, *tardbp*, *c9orf72*, and *fus*^21,31–35^. Most of these zebrafish mutants exhibit developmental phenotypes and die at the embryonic or larval stage of development. While they have provided important mechanistic insights on cellular processes and motor phenotypes, such as delayed or abnormal development of motor neurons, embryonic models fail to capture the hallmark progressive neurodegeneration that are characteristic of human ALS. Interestingly, a small subset of the genetic models generated in zebrafish develop ALS in adulthood; these models exhibit preclinical, presymptomatic, and clinical stages of the disease, which is more representative of the progression of human ALS^36–39^. Moreover, adult disease models are particularly important for investigating the role of skeletal muscle in the pathogenesis of ALS. In zebrafish and other organisms, embryonic and adult skeletal muscles exhibit distinctly different morphology, metabolism, and gene expression^40,41^.

Skeletal muscle likely contributes to the initiation and progression of ALS, although this relationship has received less attention as compared to the impact of neuronal tissues^42,43^. Bioenergetic and metabolic disturbances that affect glycolysis, oxidative phosphorylation, and lipid oxidation are known to be present in muscle cells long before the development of motor symptoms^44,45^. In addition, muscle is a trophic tissue that releases neurotrophins to support muscle stem cells (satellite cells), motor neurons, and neuromuscular junctions. Thus, disruptions in the homeostasis of skeletal muscle cells may negatively affect motor neurons as well^46^. Notably, overexpression of mutant *SOD1* that is restricted to skeletal muscle of mice is sufficient to induce ALS, demonstrating that the muscle defects present in ALS are not simply a secondary effect of motor neuron death^47^. Together, these studies highlight the possible roles that skeletal muscle plays in ALS; therefore, conceivably, skeletal muscle may serve as a therapeutic target for ALS.

Here, we sought to advance zebrafish as an adult model of ALS with efficient tools to monitor disease progression in skeletal muscle tissue. To accomplish this goal, we combined an adult-onset zebrafish model of SOD1^G93A^ ALS with an electrophysiological biomarker, electrical impedance myography (EIM), to quantitatively assess neuromuscular disease. EIM is a technique used in both research and clinical settings to evaluate neuromuscular disease status and treatment outcomes^48–55^. EIM noninvasively and quantitatively measures the bioelectrical impedance properties of muscles of interest; these measurements strongly correlate with tissue and organismal health^56^. Using a surface electrode array, a low-intensity, high-frequency electrical current is forced across a restricted area of muscle, and the consequent voltages are measured^50,56^. The relationship between the applied current and measured voltages provides quantitative metrics (i.e. resistance, reactance, and phase) that reflect the architecture and composition of the muscle (atrophy/hypertrophy, reinnervation, fibrosis, edema, and fatty infiltration)^57–60^. In rodent models of ALS, EIM has been shown to be highly sensitive to disease status and progression^48–50,61–63^. Moreover, by employing EIM in ALS clinical trials, it is possible to detect disease progression more rapidly and sensitively, in addition to the effect of therapy^50,52,53,64–69^. Thus, EIM can serve as a biomarker in both preclinical studies and clinical trials for ALS.

EIM has not been previously used to evaluate neuromuscular disease in zebrafish models of ALS. However, we have recently developed rapid and reproducible techniques for performing EIM in aging adult zebrafish^70,71^. In those studies, we compared young, healthy zebrafish to aged, sarcopenic zebrafish and found that EIM could serve as a biomarker for age-related sarcopenia. In this study, we tested the hypothesis that surface electrical impedance myography is sensitive to muscle tissue alterations observed in adult zebrafish with SOD1^G93A^ ALS. To test our hypothesis, we studied adult wildtype and ALS zebrafish at two time points. We acquired multifrequency electrical impedance data (1 kHz–1 MHz) and assessed established metrics of ALS, including spinal cord motor neuron density, the cross-sectional area of Type 1 and 2 myofibers, and motoric function. In ALS zebrafish, as compared to wildtype animals, EIM parameters at 2 and 50 kHz (phase angle, reactance, and resistance) were robust metrics that detected ALS. Moreover, EIM parameters exhibited excellent test–retest reliability in both healthy and ALS zebrafish. In sum, these findings demonstrate that EIM may be an effective tool to monitor disease progression in adult ALS zebrafish, and the overall approach identified here offers a fast, noninvasive, and reliable platform that will enable future preclinical efficacy testing of candidate therapeutics.

## Methods

### Animals

Zebrafish were housed under standard conditions (28.5 °C on a 14/10 h light/dark cycle) and fed decapsulated brine shrimp (*Artemia*) twice a day. The SOD1^G93A^ transgenic line (mi3021Tg)^36^ was obtained from Zebrafish International Resource Center (ZIRC)^72^ was outcrossed to the wildtype AB line. Sibling wildtypes were used as controls. Males were used in this study.

### Morphometric measurements

Body weight was determined by removing excess water with paper towels and weighing the animal on a tared scale. Images of whole zebrafish were captured and analyzed in ImageJ. To determine truck thickness, a line was drawn between the anterior side of the dorsal fin, where the fin meets the caudal trunk, and the anterior side of the anal fin, where the fin meets the ventral trunk (Supplementary Fig. 1).

### Western blot

Euthanized animals were bisected posterior to the dorsal fin, and a section of trunk tissue was subsequently lysed in RIPA buffer supplemented with protease inhibitors. Lysates were run on Bis–Tris gels (4%-20%) in MOPS buffer and transferred to PVDF membranes. SOD1 (Fischer Scientific PIPA130195) and HRP-secondary antibodies were used. Following antibody probing and imaging, the membrane was stained with Coomassie to verify equal protein loading.

### Electrical Impedance myography

Our surface EIM methodology for adult zebrafish has been previously described in detail, including descaling, electrode construction, and data acquisition^70,71^. Briefly, measurements were acquired from the descaled epaxial caudal musculature of anesthetized zebrafish. The dorsal fin was used as a landmark to position the electrode. The electrode array consisted of four blunt-tip needle electrodes, which were connected to the mView impedance-measuring device (Myolex®, Inc, Boston, MA, USA). The electrode was 3 mm in length (compared to the ~ 30 mm standard length of the animals in this study). The mView device was connected to a laptop running mView software (Myolex®, Inc, Boston, MA, USA) to acquire the data. A micromanipulator (World Precision Instruments, Sarasota, FL, USA) and a stereo-dissection microscope were used to control the electrode placement and its angle of contact. A pair of outer electrodes delivered alternating electrical current (400 µA, 41 frequencies between 1 kHz and 1 MHz) to the muscle while the inner pair of inner electrodes measured the resulting voltages from which impedance data are derived (resistance, reactance, and phase angle). Impedance is calculated with the following equation:

$$Z=\sqrt{{R}^{2}+ {X}^{2}}$$

where Z is the complex impedance, R is the reactance, and X is the resistance. The following formula defines phase angle (θ):

$$\uptheta =\text{ arctan }\left(\frac{\text{X}}{\text{R}}\right)$$

where X represents reactance and R represents resistance. Cell membranes, connective tissue, and adipose tissue act as capacitive elements, thus contributing to the reactance parameter, while intracellular and extracellular ionic fluids contribute to the resistance parameter. The specific frequency of the current (i.e., 1 kHz–1 MHz) also affects the impedance characteristics of the tissue being measured. Phase angle combines both resistance and reactance measurements into a single metric that has the benefit of being less affected by electrode geometry and the size of the specific tissue being measured. Generally, reduced reactance and phase values indicate smaller muscle cells and increased space between muscle fibers.

### Histology

Animals were euthanized and the trunk was severed with surgical scissors using the anal fin as a morphological landmark. The tissue was fixed in Dietrich’s fixative for several days. Subsequently, the tissue was paraffin-embedded and sectioned, and slides were H&E stained at the BIDMC Histology Core. To detect spinal cord motor neurons, consecutive 18 µm paraffin sections were obtained and stained with 0.2% Cresyl Violet (2% acetic acid, 60 mM sodium acetate) overnight. Subsequently, the slides were transferred through the following series: water, differentiation solution (2 drops of acetic acid in 95% ethanol), and 100% ethanol. After dipping in xylene, the slides were mounted in Permount™ medium.

### Myofiber and motor neuron morphometrics

Images were captured on a Keyence BZ-X710 Imaging Platform. Myofibers were analyzed using Keyence BZ-X Analyzer software as previously described^70,71^. Briefly, to determine the cross-sectional myofiber area, the automated hybrid counting tool was used to segment the myofibers in combination with manual editing with the fine edit tool to correct inaccurate segmentation. The number of myofibers analyzed per animal was ~ 400. Two blinded observers acquired the measurements. Spinal cord motor neurons were quantified using morphological criteria in Cresyl violet stained tissue sections (two consecutive 18 µm sections per animal). Brightfield Z-stack images were combined into a single image using Keyence BZ-X Analyzer software. The features used to identify motor neurons included: soma shape, size, and position, and the presence of Nissl bodies^73,74^. Sections were analyzed by two blinded observers and reported as the number of neurons in a 36 µm-thick piece of spinal cord.

### Swimming endurance

Zebrafish were fasted overnight on the day before the assay. Animals were transported to the behavioral assay room and allowed to acclimate for 2 h (temperature 28 °C). A zebrafish was placed into a glass cylinder container with 700 mL of water (28 °C). Water flow was generated using a magnetic stir bar. The water velocity was set at 5 cm/s for 1 min and then ramped up to 17 cm/s over 1 min. Videos were captured by an unblinded observer alternating the assay between WT and ALS animals. The videos were scored by a blinded observer who measured the time to exhaustion when swimming at 17 cm/s. Exhaustion was defined as being pushed by the water flow through a full counterclockwise rotation in the chamber (~ 15 cm). A mesh was placed in the chamber to prevent the animal from interacting with the stir bar. The water level above the mesh, i.e., where the fish was located, was 2 inches. All animals were given a “practice” session one week prior to data collection.

### Statistics

The data were analyzed in GraphPad Prism (version 10), RStudio (2023.12.1), and Stata (version 18). Data are presented as the mean ± standard deviation unless otherwise specified. Significance was assessed using Mann–Whitney tests or ANOVAs. ICC estimates were calculated using a single-measurement, absolute-agreement, 2-way mixed-effects model^75^.

## Results

### Adult-onset zebrafish model of G93A-SOD1 amyotrophic lateral sclerosis

**Fig. 1:**
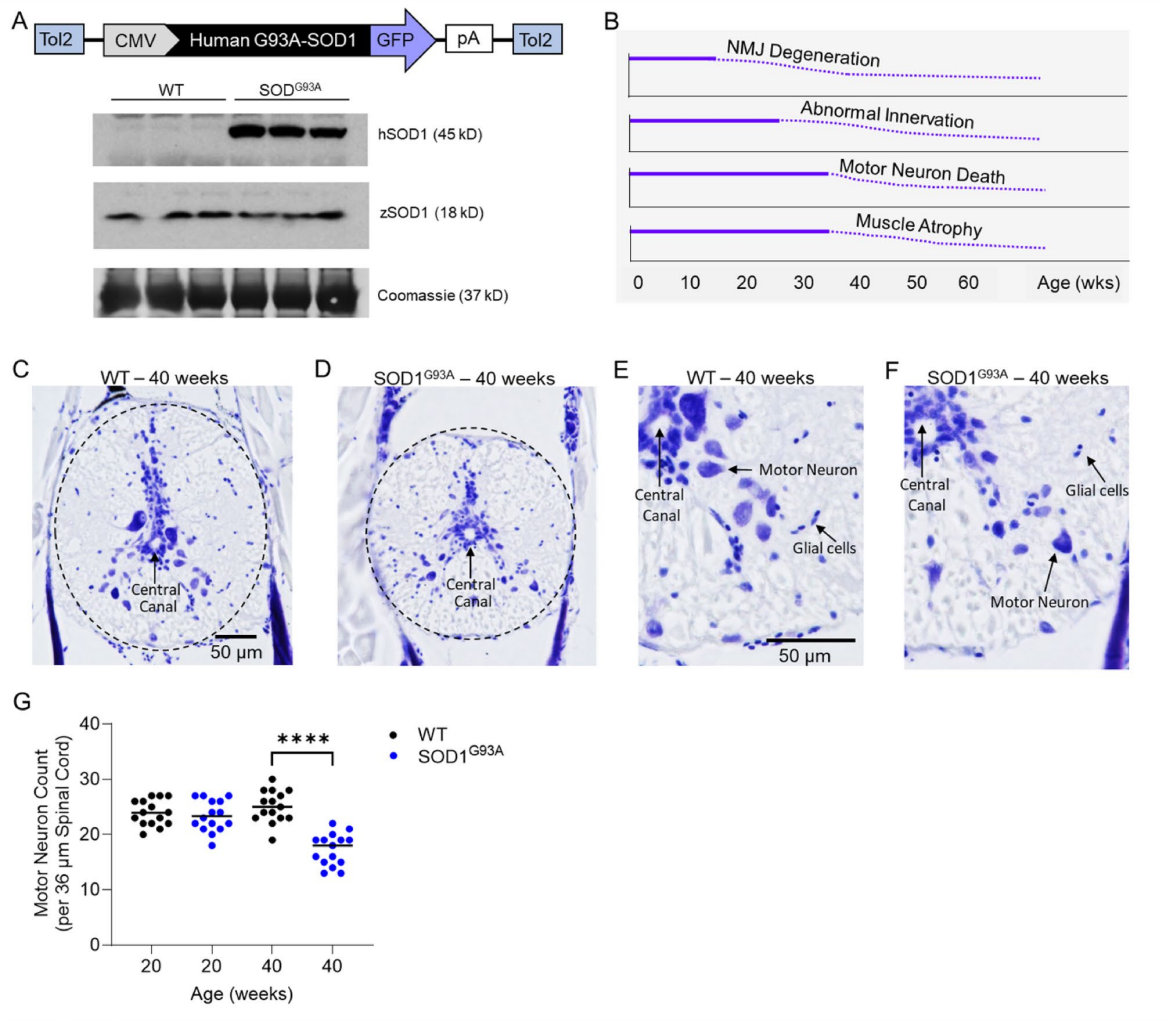
An adult-onset zebrafish model of *SOD1-G93A* amyotrophic lateral sclerosis. (**A**) The human ALS variant *SOD1*-G93A was ubiquitously overexpressed in zebrafish. Western blot for human and zebrafish SOD1 protein in lysates from adult skeletal muscle tissue. The membrane was Coomassie-stained to verify equal protein loading. See Supplementary Fig. 2 for full image of the blot. (**B**) Timeline of the phenotypic changes that occur in this adult-onset ALS model. In SOD1^G93A^ zebrafish, early pathophysiological changes in the nervous system begin to develop at the neuromuscular junction at ~ 20 weeks of age. The disease progresses between 30–60 weeks of age, with loss of motor neurons and skeletal muscle atrophy being observed by 40 weeks of age. This sequential and gradual degeneration of the neuromuscular system is characteristic of human ALS. Brightfield images of Cresyl violet stained spinal cord tissue sections from (**C**,**E**) wildtype and (**D**,**F**) SOD1^G93A^ zebrafish at the 40-week time point. Black bars = 50 µM. (**G**) Spinal cord motor neuron counts at the 20-week time point and 40-week time point in a 36 µm thick piece of spinal cord from wildtype and SOD1^G93A^ zebrafish (n = 15–16). **** = *p* < 0.0001.

In this model, zebrafish ubiquitously express the human ALS variant *SOD1-G93A* (Fig. 1A)^36^. The expression of human mutant SOD1-G93A protein is ~ twofold that of endogenous zebrafish wildtype SOD1 (Fig. 1A, Supplementary Fig. 2). In SOD1^G93A^ zebrafish, early pathophysiological changes in the nervous system begin to develop at the neuromuscular junction at ~ 20 weeks of age. The disease progresses between 30–60 weeks of age, with loss of motor neurons and skeletal muscle atrophy being observed by 40 weeks of age (Fig. 1B)^3,36^. This sequential and gradual degeneration of the neuromuscular system that begins in early adulthood and occurs over many months is characteristic of human ALS^3,36^, and makes this model, at least in some respects, superior to the very rapid decline observed in the most commonly used mouse model (B6SJL-Tg(SOD1-G93A)1Gur/J)), which occurs over about 10 weeks.

### SOD1^G93A^ zebrafish have fewer spinal cord motor neurons than wildtypes at 40 weeks but similar counts at 20 weeks

To quantify motor neurons in the spinal cords of SOD1^G93A^ and wildtype animals, cross-sectional tissue sections were obtained from animals at the vertebral level immediately posterior to the dorsal fin. Subsequently, spinal cord motor neurons were quantified in Cresyl violet stained sections at the two time points. At the 40-week timepoint, there were significantly fewer spinal cord motor neurons in SOD1^G93A^ zebrafish as compared to wildtype animals (17.3 ± 2.9 versus 25.1 ± 2.8 motor neurons, respectively; *p* < 0.0001, n = 15–16 per group, (Fig. 1C–G). By contrast, at the 20-week time point, which is very early in the pathogenesis of the disease (Fig. 1B)—when there are less intact motor neuron junctions but innervation patterns are normal, possibly due to cyclical deinnervation/reinnervation—there was no difference in the number of spinal cord motor neurons (Fig. 1G, Supplementary Fig. 3). This sequential progression of morphological changes is consistent with the disease course observed in other model organisms. For example, in a SOD1-G37R mouse model of ALS, NMJs undergo cyclical denervation and reinnervation weeks before axonal degeneration^76^. Therefore, these 2 time points were chosen to represent a non-symptomatic preclinical stage and a degenerative phase for the studies described next.

### Weight and body morphology deficits in adult SOD1^G93A^ zebrafish

**Fig. 2:**
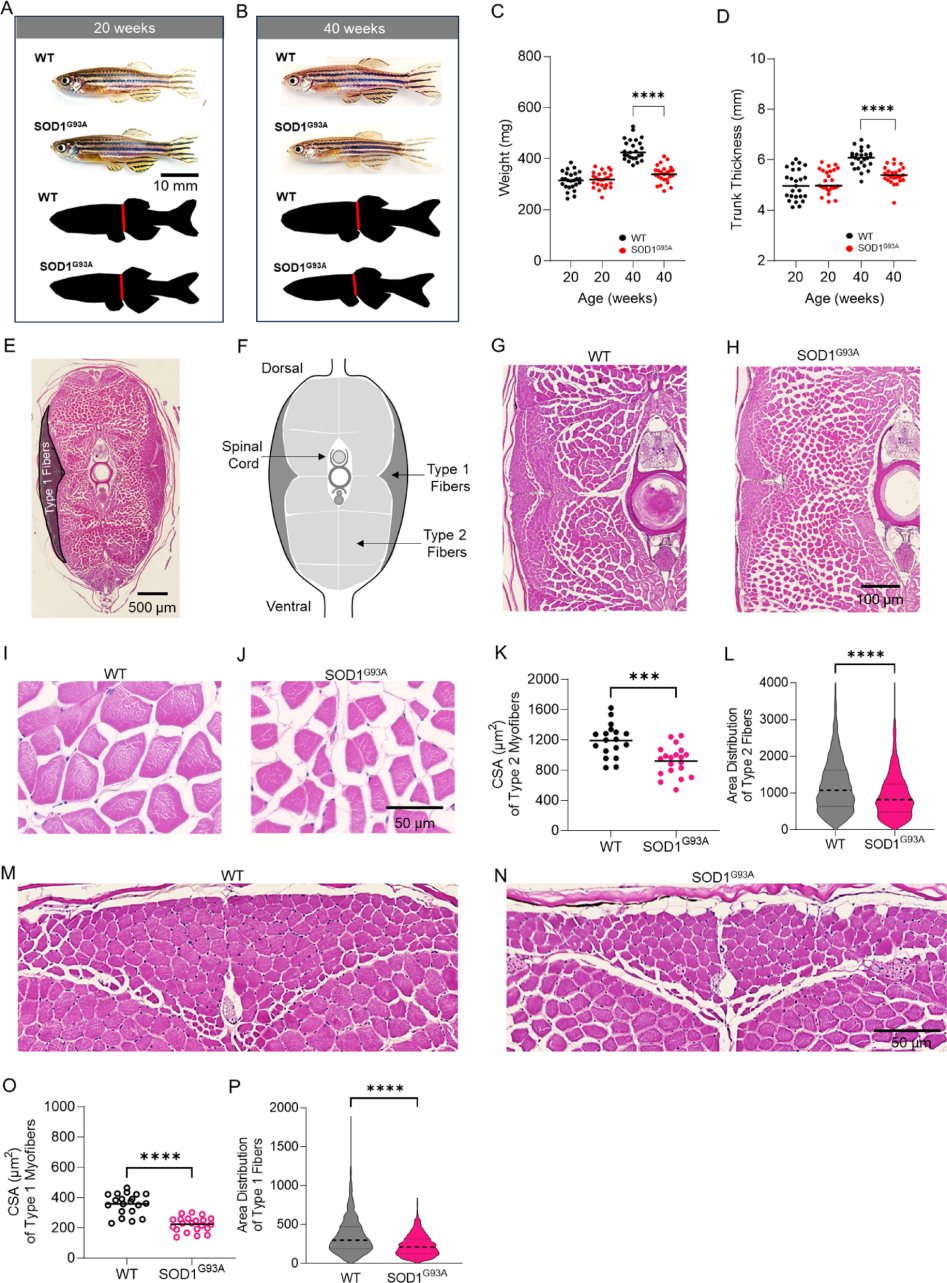
SOD1^G93A^ zebrafish exhibit decreased type 1 and type 2 myofiber cross-sectional area in the skeletal musculature. Bright field images of wildtype and SOD1^G93A^ zebrafish, and silhouettes of these images (black) at (**A**) 20 weeks of age, and (**B**) 40 weeks of age. The red line denotes the thickness of the trunk. (**C**) Animal weight and (**D**) thickness of their trunk measured at 20 and 40 weeks of age (n = 25). (**E**) Image of a hematoxylin and eosin stained tissue section obtained from a zebrafish severed at the anal fin. Black bar = 500 µM. (**F**) Drawing depicting the location of Type 1 and Type 2 fibers in the trunk lateral and caudal musculature. Hematoxylin and eosin stained tissue sections from the truck caudal musculature of (**G**,**I**) wildtype and (**H**,**J**) SOD1^G93A^ zebrafish at the 40-week time point. Black bar = 50 or 100 µM, as indicated. (**K**) Graph of the mean cross-sectional area of Type 2 fibers in the caudal musculature of SOD1^G93A^ and wildtype zebrafish at the 40-week time point (n = 18–20). (**L**) Distribution of Type 2 fiber cross-sectional areas in SOD1^G93A^ and wildtype zebrafish at the 40-week time point. Hematoxylin and eosin stained tissue sections of Type 1 fibers in the lateral musculature of (**M**) wildtype and (**N**) SOD1^G93A^ zebrafish at the 40-week time point. Black bar = 50 µM. (**O**) Graph of the mean cross-sectional area of Type 1 fibers in SOD1^G93A^ and wildtype zebrafish at the 40-week time point (n = 18–20). (***P***) Distribution of type 1 fiber cross-sectional areas in SOD1^G93A^ and wildtype zebrafish at the 40-week time point. * = *p* ≤ 0.05; ** = *p* < 0.01; *** = *p* < 0.001; **** = *p* < 0.0001.

At 20 weeks of age, i.e., when spinal cord motor neuron counts were normal (Fig. 1G), SOD1^G93A^ zebrafish appear grossly normal (Fig. 2A). However, at the same time point that we observed motor neuron loss (i.e., 40 weeks; Fig. 1G), SOD1^G93A^ zebrafish exhibit a thinner body phenotype (Fig. 2A,B). In SOD1^G93A^ zebrafish, body weight and trunk thickness were significantly decreased as compared to wildtype animals at 40 weeks of age (Fig. 2C,D): 339 ± 34 mg versus 433 ± 40 mg (*p* < 0.0001, n = 25), and 5.3 ± 0.3 mm versus 6.0 ± 0.4 mm (*p* < 0.0001, n = 25). At an earlier time-point (20 weeks of age), SOD1^G93A^ zebrafish exhibited similar body weight and trunk thickness as compared to wildtypes of the same age (Fig. 2C,D).

### SOD1^G93A^ zebrafish skeletal muscles exhibit decreased cross-sectional area of Type 1 and 2 muscle fibers

The decreased weight and trunk thickness observed at the 40-week time point in SOD1^G93A^ zebrafish suggest a loss of muscle mass (Fig. 2A–D). To quantify structural changes in skeletal muscle, histological studies and morphometric analyses were performed in cross-sectional tissue sections obtained from the trunk of 20- and 40-week-old wildtype and SOD1^G93A^ zebrafish. The architecture of caudal skeletal musculature in 20-week SOD1^G93A^ zebrafish was normal as compared to wildtype (Supplementary Fig. 4). By contrast, at 40 weeks, the caudal skeletal musculature of SOD1^G93A^ zebrafish exhibited smaller myofibers and increased space between myofibers (Fig. 2E–J). The mean cross-sectional area of Type 2 fibers was significantly decreased in SOD1^G93A^ zebrafish as compared to controls at the 40-week time point (919 ± 195 versus 1190 ± 217 µm^2^, n = 18–20, *p* < 0.001; Fig. 2K), in addition, the distribution of fiber sizes was shifted towards smaller sizes (Fig. 2L). The mean cross-sectional area of Type 1 fibers was also significantly decreased in SOD1^G93A^ zebrafish as compared to controls at the 40-week time point (223 ± 48 versus 357 ± 69 µm^2^, n = 18–20, *p* < 0.0001; Fig. 2M–O), in addition, the distribution of fiber sizes was shifted towards smaller sizes (Fig. 2P). The observed reduction in the cross-sectional area of Type 1 and 2 fibers in SOD1^G93A^ zebrafish demonstrates the presence of atrophied skeletal muscle at the 40-week time point.

### SOD1^G93A^ zebrafish exhibit reduced swimming endurance

**Fig. 3:**
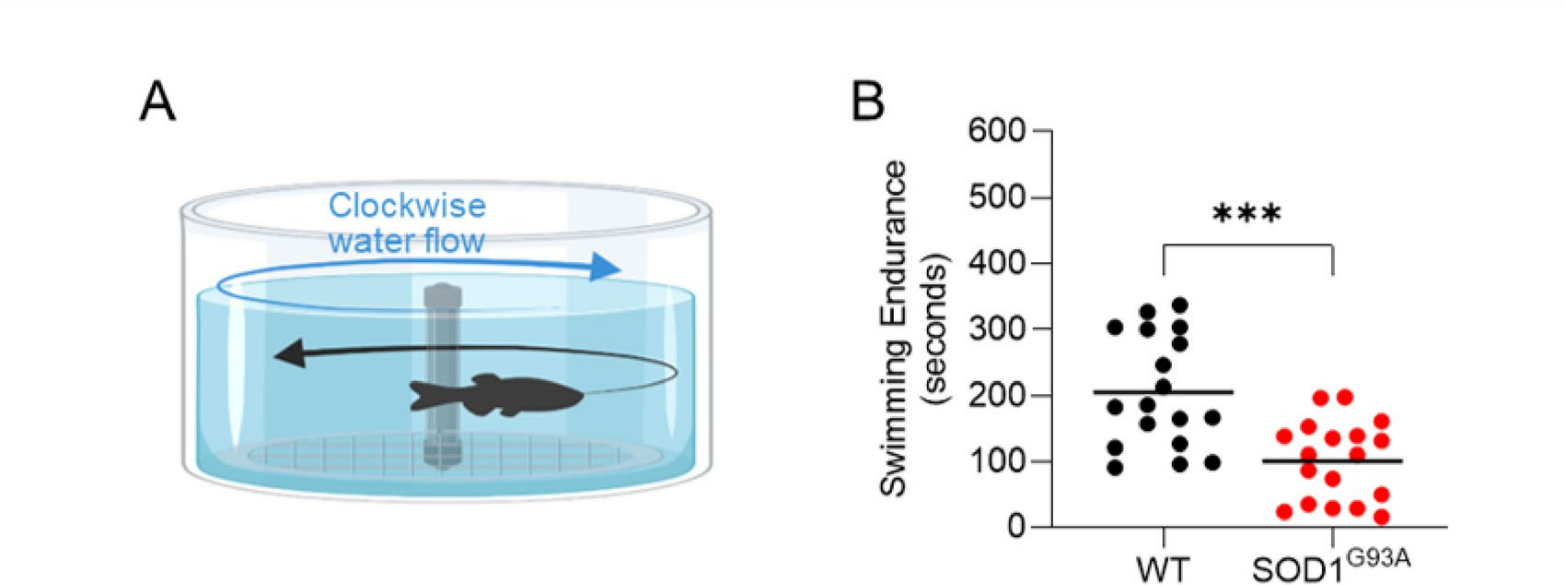
SOD1^G93A^ zebrafish exhibit reduced swimming endurance. (**A**) Depiction of the swimming endurance trial, in which zebrafish must swim against a constant flow of water. (**B**) Graph of swimming endurance time in SOD1^G93A^ and wildtype zebrafish at the 40-week time point (n = 18). *** = *p* < 0.001.

Next, we determined whether the observed loss of motor neurons (Fig. 1G) and skeletal muscle atrophy (Fig. 2K,O) in SOD^G93A^ zebrafish leads to defects in motor function at the organismal level. To assess motor function in zebrafish, animals were subjected to a swimming endurance trial in which they swam against a constant velocity of water until exhaustion (Fig. 3A). There was a significant decrease in the swimming capacity of SOD1^G93A^ animals (Fig. 3B; *p* = 0.0006), which is evidenced by a reduction in swimming time in SOD1^G93A^ animals as compared to wildtype animals (100 ± 60 s versus 205 ± 85 s, respectively). These findings indicate that the overexpression of the human *SOD1-G93A* mutation in zebrafish induces a significant defect on organismal motor function, i.e., the swimming endurance task, at the 40-week time point, which is consistent with the loss of motor neurons and muscle mass observed at this time point.

### Multifrequency electrical impedance myography detects the development of altered bioelectrical impedance properties in the caudal muscles of SOD1^G93A^ zebrafish

**Fig. 4:**
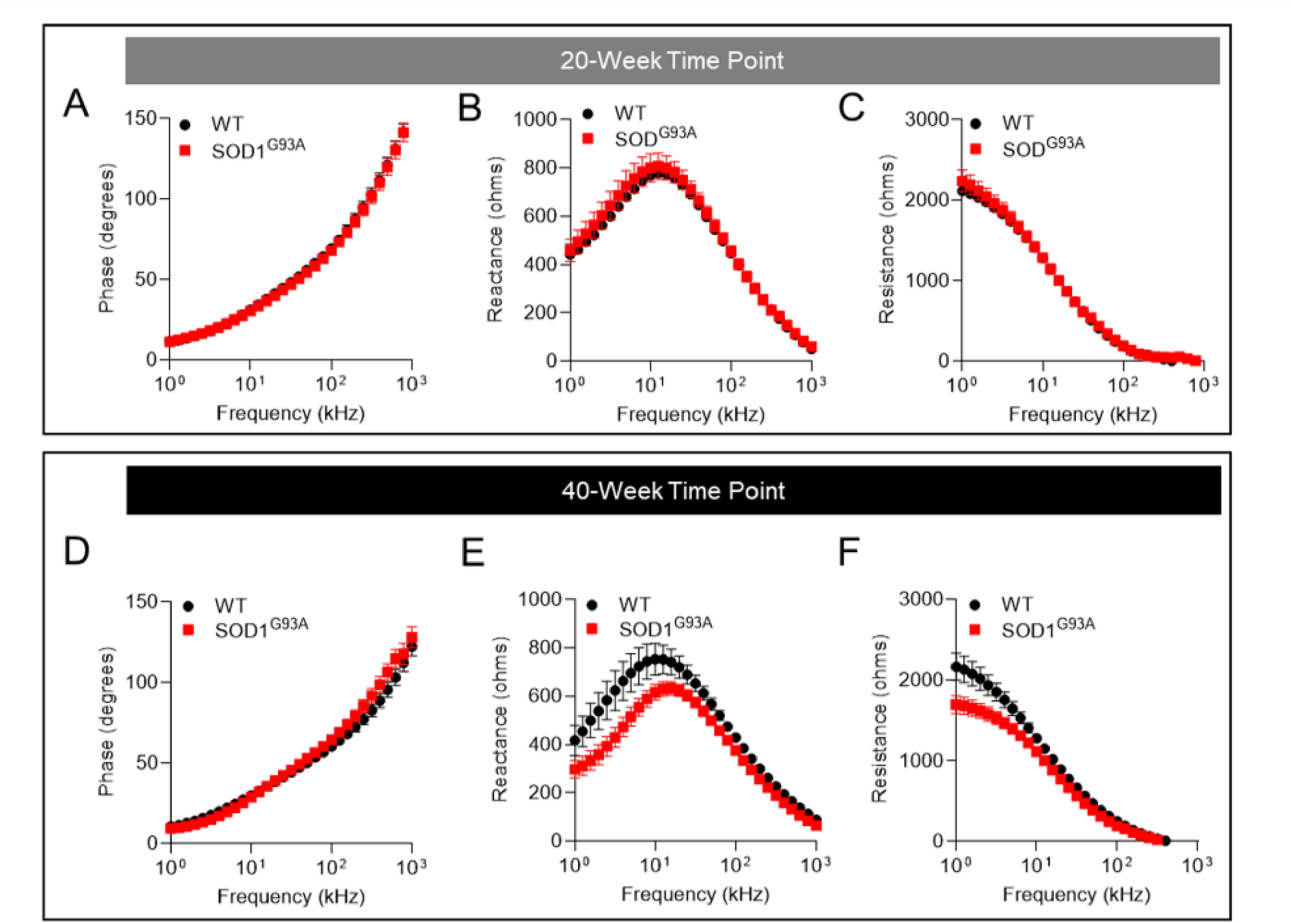
Multifrequency electrical impedance myography detects the development of altered bioelectrical impedance properties in the caudal muscles of SOD1^G93A^ zebrafish. EIM was conducted at multiple frequencies ranging from 1 to 1000 kHz, and phase angle, reactance, and resistance parameters were graphed. Graphs of (**A**) phase angle, (**B**) reactance, and (**C**) resistance in wildtype and SOD1^G93A^ zebrafish at 20 weeks of age (n = 15). Graphs of (**D**) phase angle, (**E**) reactance, and (**F**) resistance in wildtype and SOD1^G93A^ zebrafish at 40 weeks of age (n = 15–18).

We conducted EIM assessments of skeletal muscle in anesthetized zebrafish using the noninvasive methodology that we recently developed^71^. Multifrequency EIM data were collected from the dorsal caudal musculature in SOD1^G93A^ and wildtype animals that were 20 and 40 weeks of age. At 20 weeks of age, there were no overall differences in phase angle, reactance, and resistance between SOD1^G93A^ and wildtype fish (Fig. 4A–C). However, at 40 weeks of age, the multifrequency graphs of SOD1^G93A^ zebrafish exhibited a trend of decreased phase angle, reactance, and resistance at low frequencies, i.e. ≤ 50 kHz, as compared to wildtype animals (Fig. 4D–F). Together, these findings indicate that at 20 weeks of age (i.e. prior to the onset of abnormal innervation), the bioelectrical impedance properties of skeletal muscles in SOD1^G93A^ zebrafish were similar to those of wildtype fish. By contrast, at 40 weeks of age, when SOD1^G93A^ animals exhibit loss of motor neurons and muscle mass (Figs. 1G, 2K,O), an alteration in bioelectrical impedance was observed in the caudal musculature. Thus, as the disease develops, multifrequency EIM can detect the neuromuscular changes that are present in the caudal muscles of ALS zebrafish.

### Electrical impedance myography measurements at 2 and 50 kHz are reduced in SOD1^G93A^ zebrafish as the disease progresses

Next, we conducted single-frequency analyses of the EIM data at 2 and 50 kHz. An interesting aspect of conducting EIM in the zebrafish model is that, due to their thin dermis and our removal of scales, we are able to capture excellent and reliable data at these low frequencies, which predominantly reflects extracellular tissue characteristics. In mammals, data at 2 kHz is challenging to obtain with surface techniques, given the relatively greater thickness of the skin and presence of the stratum corneum that creates marked contact impedance artifacts. On the other hand, most EIM studies in mammals report data at 50 kHz because biological tissue is most electrically reactive (has the greatest charge storage capacity) at or near 50 kHz. Therefore, to be consistent with prior EIM studies, we also reported zebrafish impedance data at 50 kHz.

**Fig. 5:**
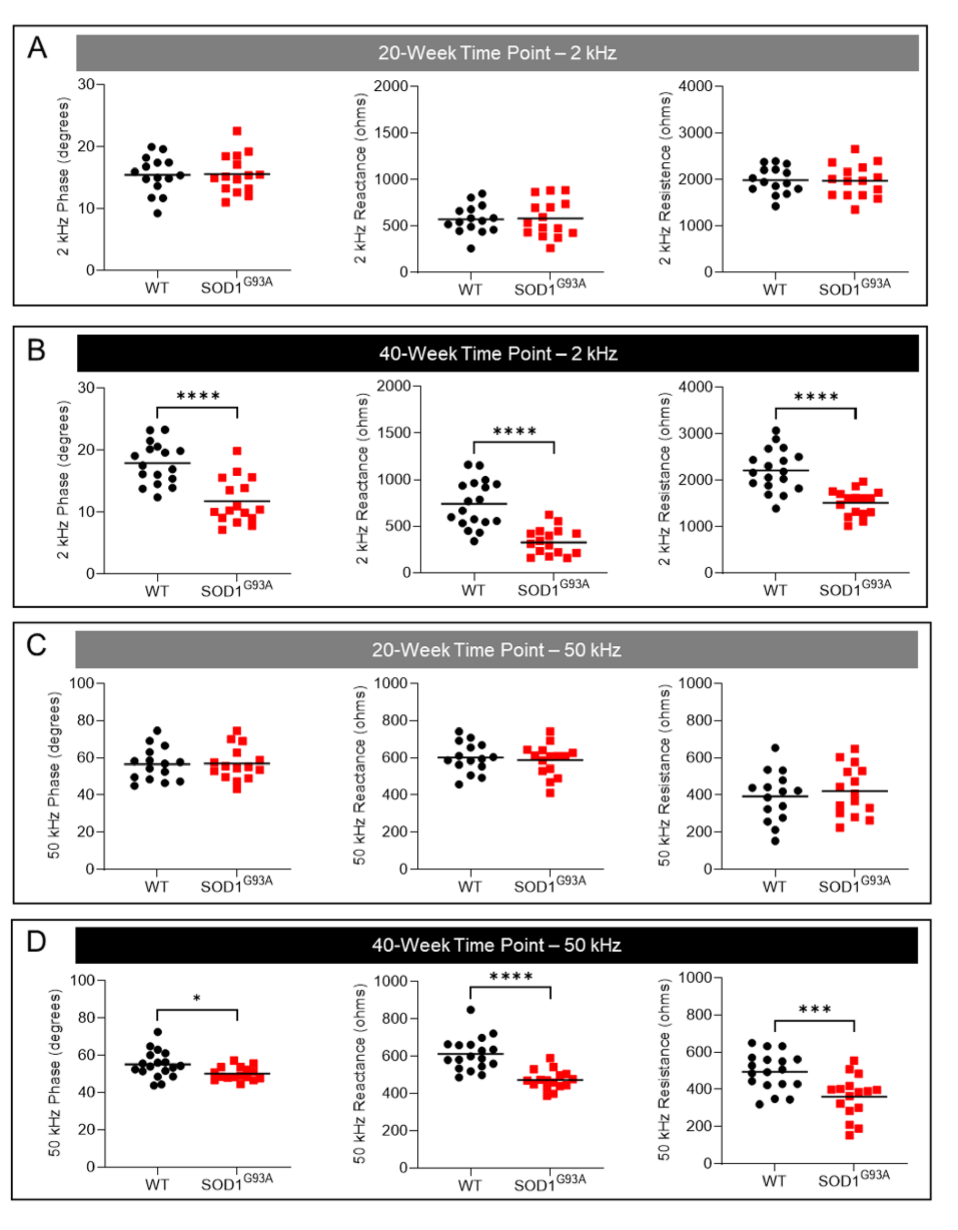
Electrical impedance myography measurements at 2 and 50 kHz are reduced as disease progresses in SOD1^G93A^ zebrafish. Analyses were conducted at 2 kHz at the (**A**) 20-week time point (n = 15), and (**B**) 40-week time point (n = 15–18). Analyses were also conducted at 50 kHz at the (**C**) 20-week time point, and (**D**) 40-week time point. * = *p* ≤ 0.05; *** = *p* < 0.001; **** = *p* < 0.0001.

At the 20-week time point, there were no significant differences in 2 kHz phase angle, reactance, and resistance between SOD1^G93A^ and wildtype animals (Fig. 5A). In contrast, at the 40-week time point, there were significant reductions in 2 kHz phase, reactance, and resistance (all *p* < 0.0001) in SOD1^G93A^ animals as compared to wildtype animals (Fig. 5B; n = 15–18): phase angle − 11.7 ± 3.6 degrees versus 17.9 ± 3.3 degrees; reactance − 340 ± 141 ohms versus 741 ± 252 ohms; and resistance − 1511 ± 277 ohms versus 2212 ± 451 ohms). For measurements acquired at 50 kHz, at the 20-week time point, there were no significant differences in phase angle, reactance, and resistance between SOD1^G93A^ and wildtype animals (Fig. 5C). However, at the 40-week time point, there was a significant reduction in 50 kHz phase (*p* = 0.01), reactance (*p* < 0.0001), and resistance (*p* = 0.0009) in SOD1^G93A^ animals as compared to wildtype animals (Fig. 5D): phase − 50 ± 3 degrees versus 55 ± 7 degrees; reactance − 488 ± 65 ohms versus 600 ± 94 ohms; and resistance − 376 ± 117 ohms versus 469 ± 126 ohms). Thus, electrical impedance myography measurements can differentiate between the skeletal musculature of healthy versus ALS zebrafish.

### Reproducibility of the EIM technique in healthy and ALS zebrafish

**Fig. 6:**
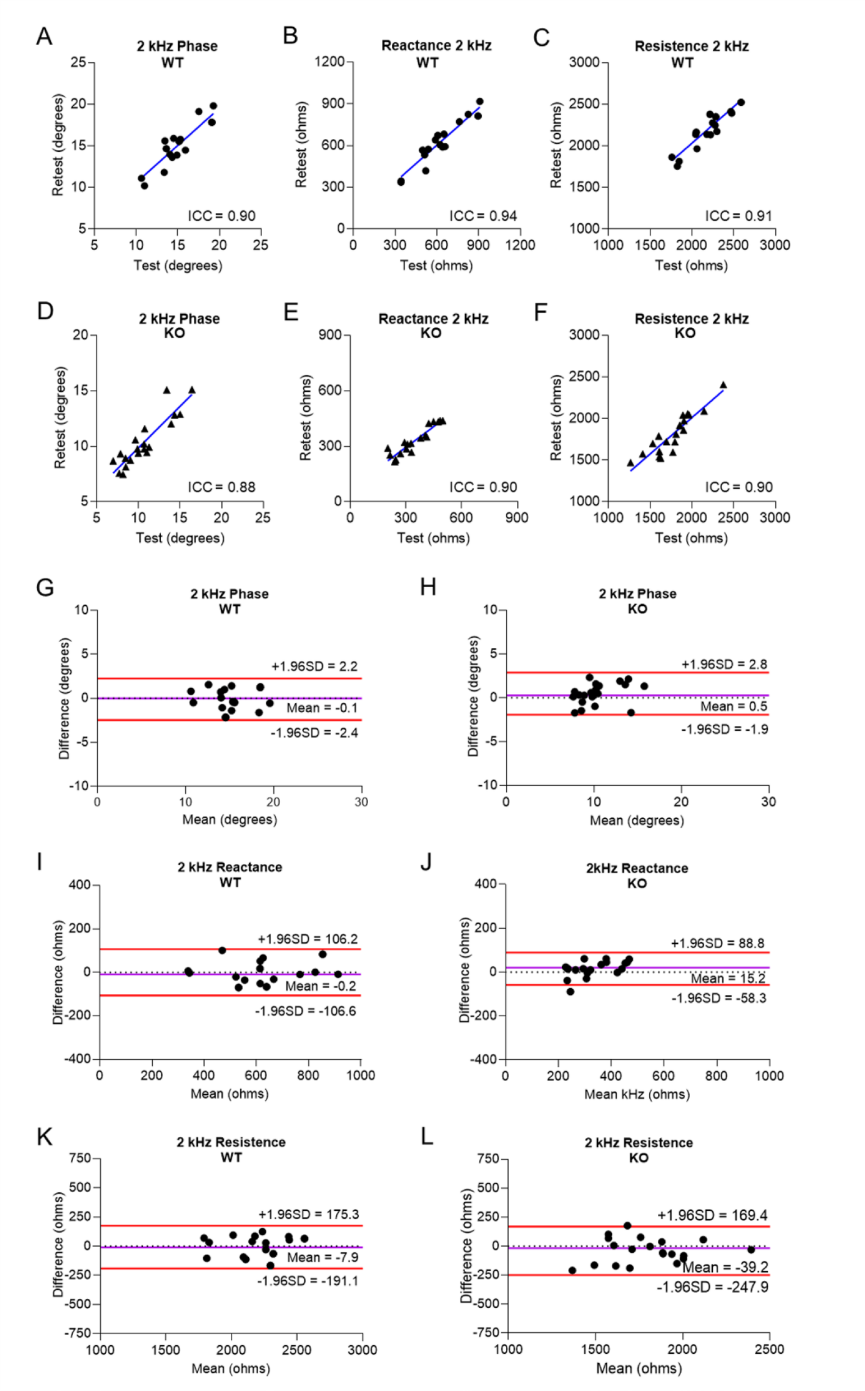
EIM exhibits good test–retest reliability in both healthy and ALS zebrafish. Analysis of the repeatability of EIM was assessed at 2 kHz by calculating the intra-class correlation coefficient (ICC). In healthy animals (n = 20), test–retest plots at 2 kHz: (**A**) phase angle, (**B**) reactance, and (**C**) resistance. In ALS animals (n = 20), test–retest plots at 2 kHz: (**D**) phase angle, (**E**) reactance, and (**F**) resistance. In healthy and ALS animals (n = 20), Bland–Altman plots of EIM parameters for 2 kHz (**G**,**H**) phase angle, (**I**,**J**) reactance, and (**K**,**L**) resistance. Red lines = the Bland–Altman 95% limits of agreement; purple line = the estimated mean difference; dotted line = the reference line of perfect average agreement (y = 0).

For assessment of technique repeatability, after an initial set of measurements was acquired, the animal was removed from the setup, placed back on the setup, and the measurements were repeated. To evaluate test–retest reliability, the intraclass correlation coefficient (ICC) between test and retest measurements was calculated for each EIM parameter (Table 1; n = 20). In healthy animals, the ICC for phase, reactance, and resistance was 0.90, 0.94, and 0.91 (*p* < 0.001), respectively (Table 1, Fig. 6A–C). In ALS animals, the ICC for phase, reactance, and resistance was 0.88, 0.90, and 0.90 (*p* < 0.001), respectively (Table 1, Fig. 6D–F). EIM data from repeated tests were depicted in Bland–Altman plots, which display the means against their respective paired differences (Table 1, Fig. 6G–L). The variance of the differences was similar across the range of measured values for each EIM parameter and in both healthy and ALS zebrafish (Fig. 6G–L). In sum, EIM exhibited excellent test–retest reliability in both healthy and ALS zebrafish.

**Table 1:** Repeatability analyses at 2 kHz.

		Phase (°)	Reactance (Ω)	Resistance (Ω)
Healthy zebrafish (n = 20)^†^	ICC^††^	0.90	0.94	0.91
	*p*-Value^††^	< 0.001	< 0.001	< 0.001
	Bias^†††^	− 0.09	− 0.22	− 7.93
	95% Limits of Agreement^†††^	− 2.4 to 2.2	− 106.6 to 106.2	− 191.1 to 175.3
ALS zebrafish (n = 20)^†^	ICC^††^	0.88	0.90	0.90
	*p*-Value^††^	< 0.001	< 0.001	< 0.001
	Bias^†††^	0.46	15.28	− 39.25
	95% Limits of Agreement^††^	− 1.9 to 2.8	− 58.3 to 88.8	− 247.9 to 169.4

ICC: intra-class correlation; SEM: standard error of the mean.

^†^For assessment of technique repeatability, after an initial set of measurements was acquired, the animal was removed from the set-up, placed back on the set-up, and the measurements were repeated.

^††^Single-measurement, absolute-agreement, 2-way mixed-effects model.

^†††^Bland–Altman analyses.

## Discussion

The initiation and progression of ALS involves multiple cell types and tissues, including a potentially causal role for the skeletal muscle system itself^42,43^. Despite our knowledge of its etiopathogenesis, ALS remains an incurable disease, with only minimally effective therapies to slow disease progression except in a few isolated genetic forms of the disease^3^. To accelerate the discovery of novel therapeutics to treat ALS, there is a need for the development of new ALS platforms that incorporate alternative animal models^14,18–20^. In this study, we sought to advance zebrafish as an alternative model of ALS with efficient tools to detect disease and measure its progression in skeletal muscle tissue. Therefore, we first rigorously validated the hallmark phenotypic changes that were reported in this transgenic line to confirm the findings of the prior study^36^, and next evaluated the ability of noninvasive electrophysiological biomarkers, i.e., EIM, to detect disease in an adult-onset zebrafish model of SOD1^G93A^ ALS.

In this study, surface electrical impedance myography was applied to anesthetized adult ALS zebrafish and control animals at 2 time points. At 20 weeks of age, body morphometrics, spinal cord motor neuron numbers, and skeletal muscle mass were normal in SOD1^G93A^ zebrafish (Figs. 1G, 2C,D, and Supplementary Fig. 3–4). Concordantly, the bioelectrical impedance properties of their skeletal muscles at this time point were also normal and similar to those of wildtypes (Figs. 4A–C, 5A,C). By contrast, at 40 weeks of age, SOD1^G93A^ animals exhibit reduced weight, loss of motor neurons, type 1 and 2 myofiber atrophy, and decreased capacity for endurance swimming (Figs. 1G, 2C,K,O, 3B). Application of the EIM device to the surface of the caudal trunk of SOD1^G93A^ animals at this time point detected a significant reduction in phase angle, reactance, and resistance (Figs. 4D–F, 5B,D) at lower frequencies. It should be noted that EIM measures muscle conductance in a small region of interest covered by the small electrode array. In the case of zebrafish, our measurements are restricted to the 3 mm distance, which is small in proportion to the entire body size (~ 30 mm). Thus, body weight or size of the animal per se does not impact EIM measurements; rather, the measurements reflect the size of the muscle cells in the measured tissue region. For example, young zebrafish weigh significantly less than aged zebrafish, ~ 33% less, and have higher 2 kHz phase angles (10.7 ± 1.5 versus 5.3 ± 2.1 degrees; *p* = 0.001) because their trunk muscles have larger myofibers as compared to aged zebrafish^70^. By contrast, in the present study, diseased ALS animals weigh less than healthy animals but have lower 2 kHz phase angles due to muscle atrophy in diseased animals. These findings demonstrate that EIM is not simply a metric of body weight but rather muscle tissue composition. In sum, as the disease develops, surface EIM can robustly detect the neuromuscular changes that are present in the muscles of ALS zebrafish. To assess the reliability of our EIM technique in healthy and ALS zebrafish, we conducted ICC and Bland–Altman analyses (Table 1 and Fig. 6) on repeated measurements on the same fish. The EIM methodology exhibited excellent reproducibility in both healthy and ALS zebrafish.

One unexpected finding of interest is that the observed differences in EIM values between wildtype and ALS fish were greatest at very low frequencies (e.g., 2 kHz) as compared to most of the rodent and human work, where the focus has been on higher frequencies (typically 50 or 100 kHz). This difference between wildtype and ALS fish is most obvious in the resistance values, which show an increasing separation as the applied current frequency decreases (Fig. 4F). There is one practical difference to explain this species difference: namely, it is very straightforward to obtain high-quality data at even low frequencies in zebrafish, which is not the case in humans or mice. This is because the interface between the electrodes and mammalian skin creates what is termed “contact impedance,” which can impact the data up to frequencies of 20 or 30 kHz in mice or humans. Perhaps because in zebrafish there is no stratum corneum, we can obtain virtually artifact-free data at very low frequencies. The second reason may relate to the basic bioimpedance concept that applied electrical current remains wholly extracellular at low frequencies. Stated another way, low-frequency current (such as 2 kHz current) will simply flow through the relatively empty interstitial space in the muscle, as captured in Fig. 2I,J. The tissue resistance would be expected to be lower in the ALS animals simply because there is an expanded extracellular space. In human or murine disease, we typically see a higher density of smaller myocytes and not a dramatic reduction in myofiber density as observed here.

Finally, our work addresses an outstanding question regarding the nonsignificant swimming data previously reported in this specific SOD1^G93A^ mutant line. A surprising finding in the prior SOD1^G93A^ zebrafish publication by Sakowski et al. was that even though histology demonstrated a reduction in the number of intact NMJs and innervation pattern defects, single timepoint swim speed and the rate of decline between 10 to 60 weeks were not significantly different, although the data consistently trended lower in ALS animals^36^. ALS animals tended to spend more time at rest, which was significant at the last timepoint studied (60 weeks). One interpretation of these findings is that the trend toward slower swim velocity in SOD1^G93A^ mutants was driven by increased time spent not moving rather than reduced velocity, which may indicate that SOD1^G93A^ zebrafish fatigue faster. However, this unexpected result remained unresolved until now. Our study used the endurance swimming assay and demonstrated that motor function defects are present in SOD1^G93A^ zebrafish (Fig. 3). We demonstrate a significant reduction in endurance at 40 weeks in SOD1^G93A^ zebrafish as compared to WTs, which resolves the absence of significant swim behaviors reported in the prior publication. Importantly, we also found that the application of noninvasive EIM robustly detected disease at this same time point.

### Electrophysiological biomarkers of ALS in preclinical and clinical studies

The use of impedance-based measures has been well-established in both rodent models and humans with ALS. The electrophysiological biomarkers acquired provide a sensitive metric to monitor ALS disease status, progression, and response to therapy^48–50,52,53,61–69^. However, rodent models are not as amenable to high-throughput studies as the zebrafish. Successfully conducting surface electrical impedance myography in ALS zebrafish combines a fast, noninvasive, and quantitative physiological tool with the experimental tractability of zebrafish. Thus, this study opens a path toward comprehensively testing a large array of zebrafish models carrying different ALS variants, such as *TARDBP*, *C9ORF72*, and others. Moreover, EIM in zebrafish may assist in the functional testing of the numerous genetic variants that have been associated with ALS via genome-wide association studies^77^.

### The SOD1^G93A^ model of ALS in zebrafish: a disease model that more closely mimics the gradual and sequential clinical deterioration characteristic of human disease than the SOD1^G93A^ mouse

Though not as numerous or as well studied as murine models of ALS, the number of genetic zebrafish models of ALS are growing and include *sod1*, *tardbp*, *c9orf72*, and *fus* models^21,31–35^. These models recapitulate key features of human ALS with defects including motor neuron axonopathy, abnormal neuromuscular junctions, mitochondrial alterations, protein aggregation, muscle degeneration, motor neuron loss, and locomotion deficits^21,31–35^. Many of the zebrafish ALS mutants are embryonic lethal^36–39^. While early developmental models have revealed fundamental insights, they do not capture the progressive neurodegeneration characteristic of human ALS. Moreover, adult disease models are particularly relevant for investigating skeletal muscle-specific mechanisms in ALS, given the fundamental differences between embryonic and adult muscles. The *SOD1*^*G93A*^ zebrafish model used in this study is an adult-onset model of ALS, accordingly it offers a translationally relevant platform for studying disease progression and therapeutic interventions^3,36^.

In SOD1^G93A^ zebrafish, early pathophysiological changes in the nervous system are just beginning to develop at the neuromuscular junction at ~ 20 weeks of age. The disease progresses between 30–60 weeks of age, with loss of motor neurons and skeletal muscle atrophy being observed by 40 weeks of age (Figs. 1G, 2K,O) and premature death beginning at ~ 18 months of age. Importantly, the slower rate of disease progression in the zebrafish SOD1^G93A^ model, occurring over many months, also provides a longer diagnostic and treatment window as compared to that of the commonly used *B6SJL-Tg(SOD1-G93A)1Gur/J)*) murine model, which spans just 10 weeks. Another notable advantage of this zebrafish ALS model is that the level of SOD1^G93A^ mutant protein expression (Fig. 1A) closely mirrors that observed in ALS patients, in contrast to the supraphysiological levels often required to elicit ALS disease phenotypes in some mouse models^13–15,20,36^. Animal models that express supraphysiological levels of mutant protein and exhibit a rapid disease course may fail to faithfully recapitulate the cumulative, progressive processes underlying neuromuscular degeneration; these factors could have contributed to the limited translatability of ALS drug discovery efforts from mice to humans. To conclude, this specific zebrafish SOD1^G93A^ model, combined with EIM assessment, creates a clinically relevant platform that mirrors the progression of human ALS of disease and could be leveraged to evaluate potential treatments for ALS using noninvasive electrophysiological biomarkers.

### Additional valuable features of the zebrafish for modeling ALS and other neuromuscular diseases

Many features of the zebrafish make this organism an attractive preclinical animal model^19,78–82^. This is because zebrafish disease models not only mirror the pathogenesis of human diseases through conserved molecular mechanisms, but also offer significantly greater scalability than mouse models. Zebrafish are small (3 cm) vertebrates that produce hundreds of embryos every week in a small, inexpensive-to-house footprint; thus, hundreds of disease-related genes or small molecules can be evaluated in a whole organism. A unique advantage of the zebrafish model is the fact that muscle fiber types are completely separated anatomically, with Type 2, fast-twitch fibers occupying the dorsal and ventral regions and Type 1, slow-twitch fibers occupying the lateral regions (Fig. 2). This greatly simplifies the assessment of therapies that impact fiber types differently, which has been a challenge in mammalian models. Finally, the conservation of disease-related proteins and pathways between humans and zebrafish ensures that drug targets are also conserved, thereby enabling effective therapeutic discovery. Indeed, in the past decade, ~ 20 candidate therapeutics entered clinical trials that were discovered in zebrafish platforms, highlighting the translatability of zebrafish findings to humans^78–82^. These features make zebrafish ideally suited to enable drug discovery for ALS.

### Skeletal muscle as a therapeutic target for ALS

Neuronal tissues play a central role in ALS; however, skeletal muscle likely has a part to play in the disease etiopathogenesis, although this relationship is less well studied than the impact of neuronal tissues. Skeletal muscle is approximately 40% of body mass in healthy individuals, and maintaining muscle mass quantity and quality through the lifespan has important implications in the prevention of neurological and neurodegenerative diseases^83^. A reciprocal and supportive crosstalk exists between muscle fibers and motor neurons. The health of neuromuscular junctions and motor neurons is supported by trophic factors released from skeletal muscle. On the other hand, innervation supports the maintenance of muscle fiber size. Multiple genes associated with ALS (*SOD1*, *TARDBP*, etc.) have known roles at the neuromuscular junction, and, regardless of the genetic cause, an early feature in the disease is neuromuscular junction defects. In addition, skeletal muscle may contribute to disease pathogenesis through mechanisms beyond denervation. Alterations in muscle metabolism have also been proposed as a common pathological pathway in ALS^84,85^. For example, TDP-43 aggregates are present in muscle biopsies in addition to defects in glucose oxidation, and TDP-43 overexpression in skeletal muscle of mice alters glucose homeostasis and fat deposition^86–88^. Thus, dysfunctional mechanisms residing within skeletal muscle cells may contribute to the disease.

Additional evidence supporting the concept that skeletal muscle plays an active role in modulating ALS pathogenesis is derived from studies using tissue-restricted expression of ALS mutations. Among the known genetic variants associated with ALS, the majority of tissue-restricted transgenic murine lines created to date used *SOD1* variants^89^. Cell and tissue-specific *SOD1-G93A* and *-G37R* mutant murine lines demonstrated that motor neuron-restricted expression was not sufficient to induce ALS, although there are some conflicting reports; nonetheless, skeletal muscle-restricted expression induced the disease^90–93^. These findings suggest that causal pathologic mechanisms of ALS reside within skeletal muscle cells, and, potentially, avenues for therapeutic intervention to treat ALS also reside there. Indeed, in *SOD1-G93A* global mice, muscle-restricted expression of insulin-like growth factor-1 stabilized neuromuscular junctions, increased satellite cell activity, preserved myofibers and motor neurons, and remarkably prolonged lifespan by 1 month^94^. In another study in *SOD1-G93A* global mice, pharmacological activation of the purogenic receptor P2X7, which is highly expressed in skeletal muscle, increased satellite cell differentiation, preserved innervated endplates, and prevented myofiber atrophy^95^. These and other proof-of-concept studies illuminate the therapeutic potential of modulating skeletal muscle metabolism and muscle differentiation to treat ALS^96^. Accordingly, future tissue-specific studies are warranted. The zebrafish, with its inexpensive husbandry and experimental tractability, combined with its established toolbox for cell and tissue-specific genetic perturbations, offers a facile approach for dissecting the roles of skeletal muscle in ALS.

### Limitations and future directions

This study has the following limitations. First, we tested only one type of EIM electrode array. Probe size and electrode distance affect the penetration of the applied current into the tissue being evaluated and potentially other tissues^97,98^. The tissue region and tissue depth measured affect the reproducibility of the data. Nevertheless, the electrode array used in this study exhibited good reliability in both healthy and ALS zebrafish. Future work will optimize the probe array to improve reliability further. Second, in this study, we evaluated intra-rater reliability and did not assess inter-rater reliability. However, in previous work, we demonstrated high inter-rater reliability of the EIM technique in healthy zebrafish^70,71^. Third, we assessed one muscle region in this study. The epaxial caudal musculature of the trunk was selected because of the ease of repeatably positioning the electrode array using a reliable morphological landmark (i.e., the dorsal fin). Moreover, we did not assess muscle anisotropy (the transverse measurement minus the longitudinal measurement), which has been shown to decrease in tissues with increased fat and connective tissue^97^. Future work will investigate anisotropy in SOD1^G93A^ zebrafish to determine if it is a useful metric to evaluate neuromuscular disease in ALS zebrafish. Fourth, another limitation of this work is that we did not attempt to track individual fish over many months to fully characterize the rate of change in our EIM and behavioral parameters as ALS progressed; such an approach will be needed when we use this methodology in actual therapeutic efficacy assessment. Our prior studies assessing temporal EIM measurements in zebrafish demonstrated the feasibility of collecting multiple data points on the same zebrafish over time, albeit those studies were in young, healthy animals^71^. In future studies, we will perform longitudinal assessments in ALS and control animals to identify subtle early changes in disease progression and to delineate the specific noninvasive metrics—such as motor function and/or electrical impedance myography (EIM)—that correlate with morphological alterations in the neuromuscular system over time. Indeed, the longitudinal EIM approach has proven to be very effective in studying murine models of ALS^49,99^. Finally, although beyond the scope of this study, the feasibility of conducting experiments with large numbers of subjects in zebrafish cohorts enables machine learning approaches, which benefit from large sample sizes, to interrogate EIM data at multiple different frequencies and in combination with multi-modal data (morphological metrics, motoric function endpoints, metabolomics profile, etc.).

### Impact statement

Electrical impedance myography is an electrophysiological technique for quantifying disease progression in both rodent models of ALS and patients with ALS. Our findings demonstrate that EIM is a robust, reliable, and noninvasive measure in the zebrafish ALS model. Moreover, the specific EIM metrics identified here provide efficacy markers for future therapeutic studies. In sum, by successful applying surface EIM to ALS zebrafish, we have advanced a powerful and scalable platform to accelerate the discovery of therapeutic drugs to treat ALS.

## Supplementary Information

Supplementary Information.

## Data Availability

The datasets generated during and/or analyzed during the current study are available from the corresponding author upon reasonable request.
