# Supplementary material for: Surface electrical impedance myography detects disease in an adult-onset SOD1-G93A zebrafish model of amyotrophic lateral sclerosis
Source: Sci Rep. 2025 Oct 14;15:35810. doi: 10.1038/s41598-025-19830-w (PMC12521378; doi:10.1038/s41598-025-19830-w)
Supplement: Supplementary file 1 — Supplementary Information. [file 41598_2025_19830_MOESM1_ESM.pdf]

SUPPLEMENTARY MATERIAL

Surface Electrical Impedance Myography Detects Disease in an Adult-Onset SOD1-G93A Zebrafish Model of Amyotrophic Lateral Sclerosis

Seward B. Rutkove[1,2,‡], Priyansh Shah[2,3], Laura Hevenor[3], Gaurav Tiwari[3], Dhrumil Patil[2,3], Tyler Mourey[4], Janice A. Nagy[1], and Anjali K. Nath[2,4,5,‡]

[1]Department of Neurology, Beth Israel Deaconess Medical Center, Boston, MA, 02115, USA.

[2]Harvard Medical School, Boston, MA 02115, USA.

[3]Department of Cardiology, Beth Israel Deaconess Medical Center, Boston, MA 02115, USA.

[4]Zebrafish Core Facility, Beth Israel Deaconess Medical Center, Boston, MA 02215, USA.

[5]Broad Institute, Cambridge, MA 02142, USA.

[‡]To whom correspondence should be addressed: Anjali K. Nath (anath1@bidmc.harvard.edu) and Seward B. Rutkove (srutkove@bidmc.harvard.edu)

Keywords: amyotrophic lateral sclerosis; neuromuscular disease; skeletal muscle atrophy; zebrafish; preclinical animal models; electrical impedance myography; reliability measures; biomarkers; skeletal muscle; slow twitch muscle fibers; fast twitch muscle fibers

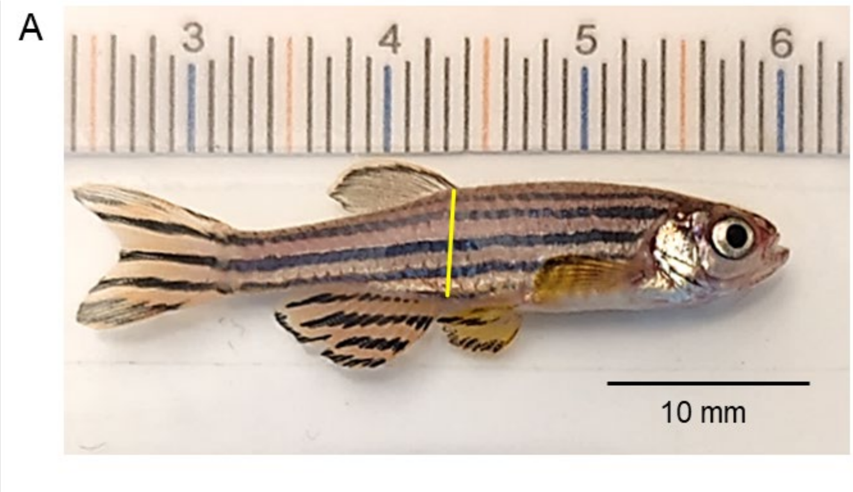

Supplementary Figure 1. Measuring trunk thickness in zebrafish. Brightfield images of zebrafish were captured to measure trunk thickness. To determine caudal truck thickness, a line was drawn between the anterior side of the dorsal fin, where the fin meets the dorsal trunk, and the anterior side of the anal fin, where the fin meets the ventral trunk (yellow line). The length was measured in ImageJ.

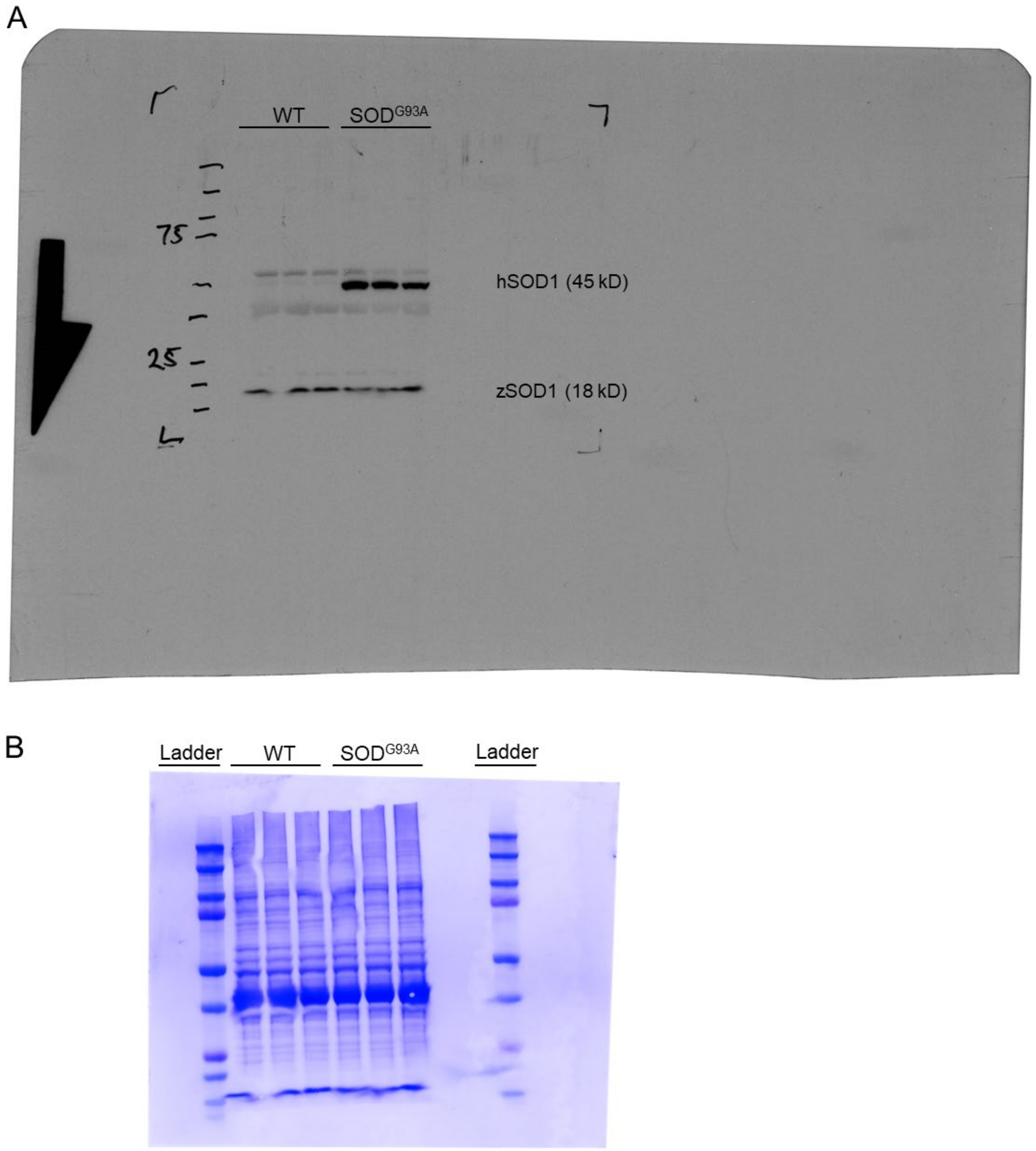

Supplementary Figure 2. SOD1 expression in WT and ALS animals. A) Image of full Western blot of zebrafish lysates showing human SOD1-G93A and zebrafish SOD1. **B)** Image of Coomassie-stained PVDF membrane.

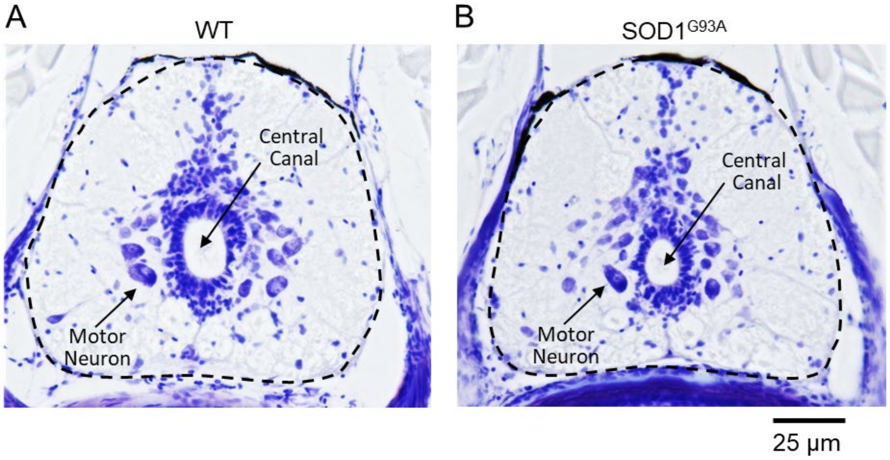

Supplementary Figure 3. Spinal cord motor neurons in wildtype and SOD1^{G93A} zebrafish at the 20-week time point. Brightfield images (merged Z-stacks) of Cresyl violet stained spinal cord tissue sections from **A)** wildtype and **B)** SOD1^{G93A} zebrafish at 20 weeks of age. Black bars = 25 µM.

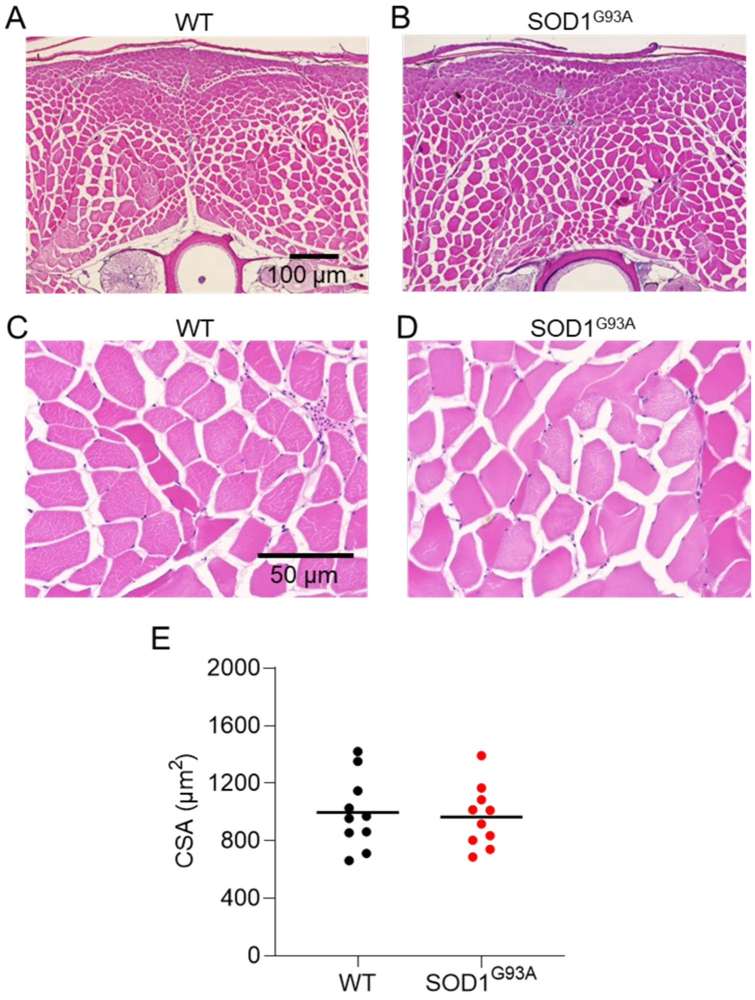

Supplementary Figure 4. SOD1^{G93A} zebrafish at 20 weeks of age exhibit normal cross-sectional myofiber area in the caudal musculature. Myofiber area was assessed in hematoxylin and eosin stained tissue sections from the truck musculature of zebrafish at the 20-week time point: **A,C)** wildtype and **B,D)** SOD1^{G93A} zebrafish. Note, the 20-week time point was chosen for "baseline" electrophysiological studies because muscle atrophy has not yet developed. Black bar = 50 or 100 μM, as indicated. **E)** Graph of the mean cross-sectional myofiber area of the epaxial caudal musculature in SOD1^{G93A} and wildtype zebrafish (n=12).
